# Supplementary material for: CRISPR-Cas9 induces large structural variants at on-target and off-target sites in vivo that segregate across generations
Source: Nat Commun. 2022 Feb 2;13:627. doi: 10.1038/s41467-022-28244-5 (PMC8810904; doi:10.1038/s41467-022-28244-5)
Supplement: Supplementary file 1 — Supplementary Information [file 41467_2022_28244_MOESM1_ESM.pdf]

Supplementary Material: CRISPR-Cas9 induces large structural variants at on-target and off-target sites *in vivo* that segregate across generations

Ida Höijer, Anastasia Emmanouilidou, Rebecka Östlund, Robin van Schendel, Selma Bozorgpana, Marcel Tijsterman, Lars Feuk, Ulf Gyllensten, Marcel den Hoed, Adam Ameur

Supplementary Figures

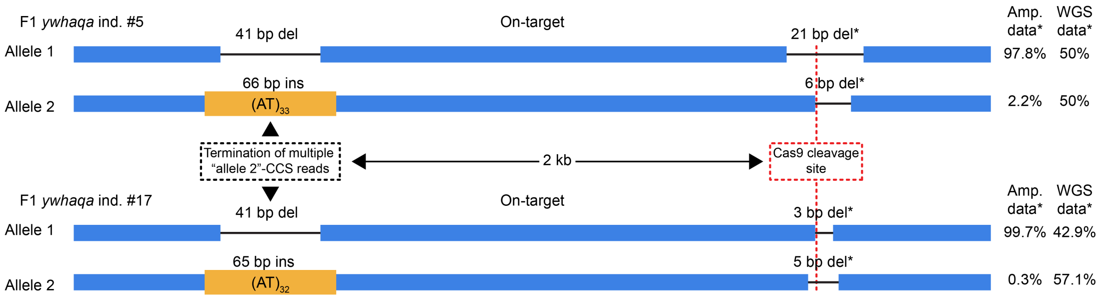

Supplementary Figure S1. Schematic figure showing the two different alleles at the on-target site in F1 *ywhaqa* individual #5 and #17. Nanopore WGS data demonstrates that the two individuals are heterozygous, with alleles 1 and 2 detected at similar frequencies. In both samples, allele 2 is heavily underrepresented in the PacBio amplicon data. A 65-66 bp insertion, consisting of an AT-dinucleotide repeat was found in allele 2 in both samples. In the PacBio amplicon data, for both samples, several CCS reads representing allele 2 terminated at this repeat insertion site.

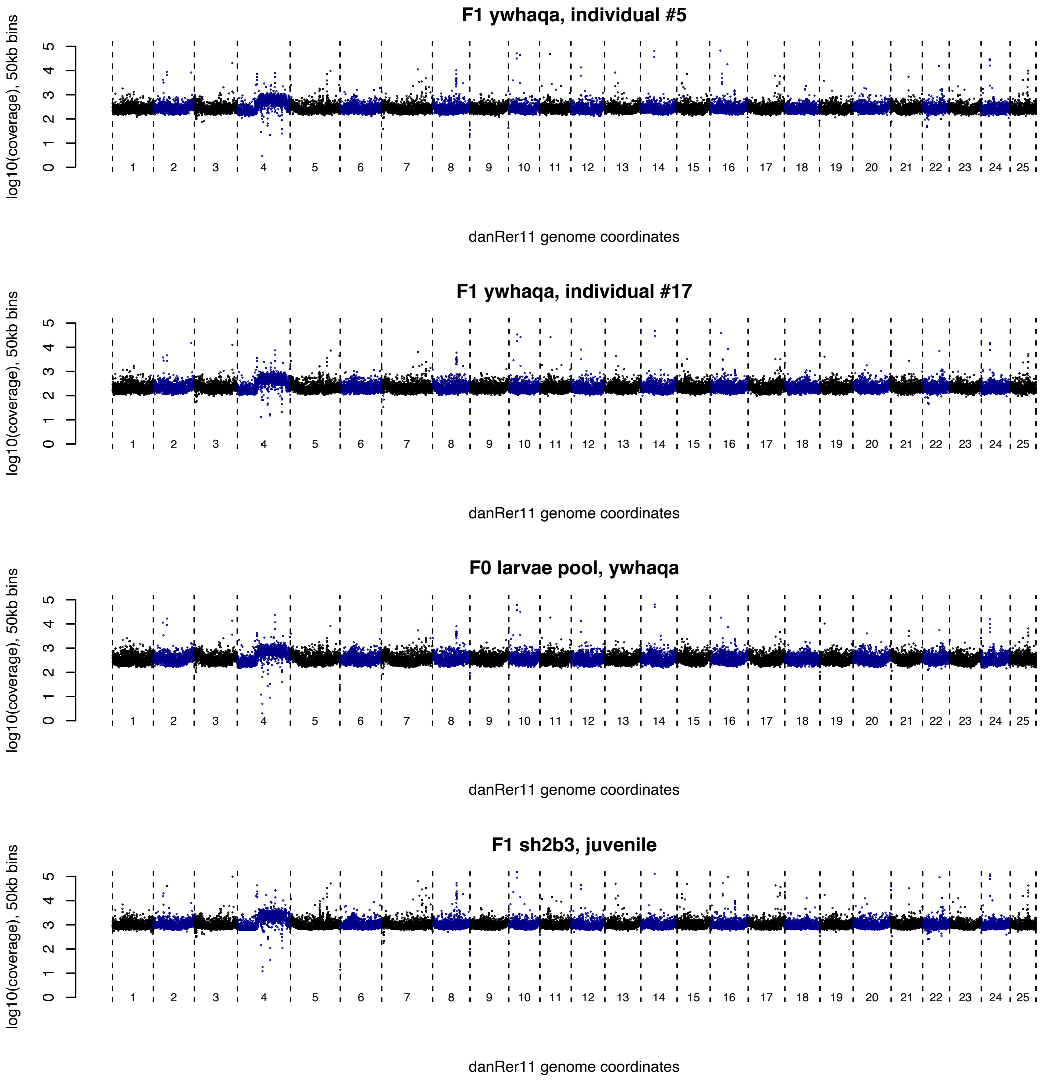

Supplementary Figure S2. Coverage distribution of nanopore whole genome sequencing data for four zebrafish samples. Each point represents the coverage in a 50 kb window. No large-scale chromosomal aberrations are observable for the F1 individuals #5 and #17 on chromosome 20, i.e., the chromosome where the *ywhaqa* on-target site is located. For comparison, nanopore WGS data was generated also for a *ywhaqa* F0 larvae pool as well as a *sh2b3* F1 individual. All four samples display a similar coverage pattern and there are no indications of large copy number variants (CNVs) being induced by CRISPR-Cas9 in these samples. A potential copy number gain signal is seen on chromosome 4, but since it occurs in all samples this signal is likely caused by general sequence differences when comparing the zebrafish samples to the GRCz11 reference.

Supplementary Tables

Supplementary Table S1. Sequences for all 23 gRNAs used in this study.

gRNA name	gRNA sequence
apoba	GGGGAGGGCTCTATCTTAGG
apobb.1	GGAGCTGACAAAGTACCAAG
apobb.2	GGATTAGCTGAGCAAGAGGA
apoc2	GGGTGTACATACCCAGTGCA
apoea	GGCTCTCTCCTGCGCGTAAG
apoeb	GAGGGGCATCAGCCTGGAAC
gckr	GGCATGTGTATTATCTGGGA
hcn4	GGAGAGCCCTCCTGGAGCCG
hcn4l	GGCCTGGATAGTGTTTAACG
insra	GGGACAGAGGCCAGCACAAG
insrb	GACCAACATCACGCGGGGAG
kcnv2a	GAGGAAACGCGTATGCCCAA
ldlra	GATTCACGGCAGTATCAGTG
ldlrb	GGAGCGGATTCTGCGAGCGG
lipca	GGGTTCTCCCAGACATAGCT
mmp20a	GGGATGAAAGAGGCAAGATG
nbeal2	GGTCCCCCTTCTCCCCAAGG
neo1a	GGAGCCGTCGGATACACTAG
neo1b	GGACAGAGATGCTCGGCCTG
pcsk9	GGCAGTAAAGTTGCCCCATG
sh2b3	GGGAGCGCTGGAATCCATGG
vldlr	GAGCAGTCTCAGTTCCAGTG
ywhaqa	GGCGCCTACAAGAACGTGGT

Supplementary Table S2. Cas9 cleavage sites *in vitro* for 23 gRNAs, detected by the Nano-OTS method. Coordinates and sequences are obtained from the GRCz11 zebrafish reference genome.

chr	position	gRNAname	gDNAsequence	alignSimilarity	alignScore	snvBases	indelBases	peakHeight
chr17	30718007	apoba	TGGGAGGGCTCTATCTTAGGGGG	21/80 (26.2%)	99	1	0	192
chr14	40455513	apoba	CCGCCTATGATAGAGCCCTAATT	17/80 (21.2%)	63	5	0	111
chr22	36284098	apoba	GGGGAAGGCAGTATCTTAGGGGG	19/80 (23.8%)	81	3	0	85
chr20	31273933	apobb1	GCAGCTGACAAAGTACCAAGGGG	21/80 (26.2%)	99	1	0	65
chr22	4914510	apobb1	CCTCTTGGTTCTTTGTACAGCTCT	20/80 (25.0%)	70	2	1	23
chr20	53444334	apobb2	CCATCCTCTTGCTCAGCTAATCA	21/80 (26.2%)	99	1	0	87
chr2	47454801	apobb2	CCCTCCTCTTGCTCTGCTACTCT	19/80 (23.8%)	81	3	0	24
chr16	23985170	apoc2	CCTTGCACTGGGTATGTACACAC	21/80 (26.2%)	99	1	0	122
chr19	10856069	apoea	CCCCTTACGCGCAGGAGAGAGCC	22/80 (27.5%)	108	0	0	116
chr16	23961550	apoeb	CCTGTTCCAGGCTGATGCCCCTC	22/80 (27.5%)	108	0	0	209
chr17	5878029	gckr	CCATCCCAGATAATACACATGCG	21/80 (26.2%)	99	1	0	411
chr23	30916318	gckr	TGTTTGTGTATTATCTGGAAAGG	18/80 (22.5%)	72	4	0	132
chr1	21357140	gckr	CCTTCTCAGATGATGCACATGCA	18/80 (22.5%)	72	4	0	60
chr21	23319983	gckr	CCAACCCAGATAATACACCACTT	16/80 (20.0%)	54	6	0	31
chr3	21773503	gckr	CCGTCCCAGATGATATACAACAT	16/80 (20.0%)	54	6	0	31
chr6	44274257	gckr	TCTGTGTGTATTATCTGGGGACG	16/80 (20.0%)	54	6	0	19
chr19	11525479	gckr	GACATGTATTTTATTT-------	12/87 (13.8%)	44	4	-1	10
chr21	16260203	gckr	ACACCCCAGACAGCACACATACG	15/80 (18.8%)	45	7	0	10
chr11	32265767	gckr	CCAACCCAGATAATACACCATAA	16/80 (20.0%)	54	6	0	8
chr4	32765162	gckr	ACTGCCCAGACAGCACACATACG	15/80 (18.8%)	45	7	0	8
chr5	671631	gckr	CACTCCCAGAATGTACACATGCT	17/80 (21.2%)	63	5	0	5
chr14	48008478	gckr	------CGGAGAAAACCCATGCG	12/86 (14.0%)	40	5	-1	4
chr18	1143644	hcn4	CCGCGGCTCCAGGAGGGCTCTCC	22/80 (27.5%)	108	0	0	31
chr25	29273090	hcn4l	CCACGTTAAACACTATCCAGGCG	21/80 (26.2%)	99	1	0	19
chr11	34856421	hcn4l	CCGAGTTAAACACTATCCCCAAC	17/80 (21.2%)	63	5	0	9
chr2	37570139	insra	GGGACAGAGGCCAGCACAAGGGG	22/80 (27.5%)	108	0	0	147
chr4	54914411	insra	GGGGGAGGGGGCAGCTCCA----	13/84 (15.5%)	41	6	-1	93
chr4	8537670	insra	CCGCTTGTGTTGGCCTCTGACTC	19/80 (23.8%)	81	3	0	88
chr23	14699329	insra	TGAACCGCTGCCTGCACAAGCTG	15/80 (18.8%)	45	7	0	10
chr9	8529080	insra	GGAACACAGGCGGACACAAGTGC	16/80 (20.0%)	54	6	0	8
chr22	11004684	insrb	CCGCTCCCCGCGTGATGTTGGTC	22/80 (27.5%)	108	0	0	121
chr14	15606594	insrb	GAGCCCCATCGGGGGGGGGGGGG	15/80 (18.8%)	45	7	0	85
chr10	15311488	kcnv2a	CCTTTGGGCATACGCGTTTCCTC	22/80 (27.5%)	108	0	0	19
chr3	19304777	ldlra	GATTCACGGCAGTATCAGTGTGG	22/80 (27.5%)	108	0	0	143
chr23	8189813	ldlra	CCCCACTGAAACTGCCGTGTACT	18/80 (22.5%)	72	4	0	32
chr16	9332405	ldlra	GCATAACGGCTGTATCAGTGTGG	18/80 (22.5%)	72	4	0	24
chr10	36351898	ldlra	CCTTACTGATACTGCAGTGAAAC	19/80 (23.8%)	81	3	0	16
chr19	25977945	ldlra	CCTCACTGATGTTGTCGTGAATG	18/80 (22.5%)	72	4	0	14
chr24	7925197	ldlra	CACTCACAGCAGTAACAGTGTGG	18/80 (22.5%)	72	4	0	8

chr7	30398243	lipca	CCTAGCTATGTCTGGGAGAACCC	22/80 (27.5%)	108	0	0	64
chr15	25555212	mmp20a	CCACATCTTGCCTCTTTCATCCC	22/80 (27.5%)	108	0	0	35
chr16	16478569	nbeal2	CCTCCTTGGGGAGAAGGGGGACA	21/80 (26.2%)	99	1	0	106
chr12	24373304	nbeal2	CGGCCCCCCTCTCCCAAAGGGGG	18/80 (22.5%)	72	4	0	88
chr8	47074469	nbeal2	CCACCTTGGGGGGAAAGGAGATT	17/80 (21.2%)	63	5	0	71
chr14	16385238	nbeal2	CCCCCTTGGGGAAATGGGGTGTT	16/80 (20.0%)	54	6	0	67
chr19	44806008	nbeal2	CCACCTTGGGGAGAAGCGGGGCA	19/80 (23.8%)	81	3	0	57
chr24	13828273	nbeal2	CACACTTGGGGAGCAGGGGGCCT	17/80 (21.2%)	63	5	0	56
chr18	14204547	nbeal2	GCACCCCCTTTCCCCCCAGGCGG	17/80 (21.2%)	63	5	0	35
chr10	1890600	nbeal2	GGTCCACCATCTTCCCAAGGAGG	19/80 (23.8%)	81	3	0	32
chr3	35685641	nbeal2	ATACCCCCCTACCCCCAAGGGGG	16/80 (20.0%)	54	6	0	22
chr20	24396692	nbeal2	CCCCCTTTGGGGGATGGGGTGGG	15/80 (18.8%)	45	7	0	19
chr6	23360436	nbeal2	CGTCCCCCTGTCTCCCCAGGGCGG	20/80 (25.0%)	70	2	1	14
chr14	11142437	nbeal2	CCACCTGGGGGGGATGGGGGGAG	16/80 (20.0%)	54	6	0	11
chr18	30359856	nbeal2	CCCCCTTGGGGAGAGGGTTCATA	16/80 (20.0%)	54	6	0	6
chr5	13924978	nbeal2	CCACCTTTGGGATATGGGGGAAC	18/80 (22.5%)	72	4	0	5
chr7	53879247	neo1a	GGAGCCGTCGGATACACTAGCGG	22/80 (27.5%)	108	0	0	70
chr2	33150097	neo1a	CCTTTAGTGTATCAGGCGGCTCT	18/80 (22.5%)	72	4	0	6
chr25	2899718	neo1b	GGACAGAGATGCTCGGCCTGTGG	22/80 (27.5%)	108	0	0	36
chr20	7399763	pcsk9	GGCAGTAAAGTTGCCCCATGTGG	22/80 (27.5%)	108	0	0	226
chr20	19914504	pcsk9	CAACAAGGATCAACATTAGTGA	14/80 (17.5%)	40	7	0	29
chr14	18273989	pcsk9	CCGAATGGGG-AACTTTACTGA	18/81 (22.2%)	60	2	1	14
chr24	27308362	pcsk9	CCGAATGGGG-AACTTTACTGA	18/81 (22.2%)	60	2	1	8
chr17	26877631	pcsk9	GTCAGTAAAGTT-CCCCATTCGG	19/81 (23.5%)	65	2	1	6
chr12	16562148	pcsk9	CCGAATGGGG-AACTTTACTGA	18/81 (22.2%)	60	2	1	6
chr20	11843066	pcsk9	CCGAATGGGG-AACTTTACTGA	18/81 (22.2%)	60	2	1	4
chr5	9624697	sh2b3	CCGCCATGGATTCCAGCGCTCCC	22/80 (27.5%)	108	0	0	49
chr15	31598137	sh2b3	GGGAGCGCAGGAATCCATGGATG	20/80 (25.0%)	90	2	0	34
chr10	20539546	sh2b3	CTAAACACTGGAATCAATGGAGG	16/80 (20.0%)	54	6	0	10
chr10	15269480	vldlr	GAGCAGTCTCAGTTCCAGTGTGG	22/80 (27.5%)	108	0	0	22
chr5	3544150	ywhaqa	GTGGCCTACAAGAACGTAGTGGG	19/80 (23.8%)	81	3	0	167
chr20	29566888	ywhaqa	GTCGCCTACAAGAACGTGGTCGG	21/80 (26.2%)	99	1	0	73
chr17	32503336	ywhaqa	GTTGCCTATAAGAACGTGGTGGG	19/80 (23.8%)	81	3	0	61
chr6	52216272	ywhaqa	GTGGCCTACAAGAATGTGGTAGG	19/80 (23.8%)	81	3	0	28
chr16	24684272	ywhaqa	GTGGCTTACAAGAACGTGGTGGG	19/80 (23.8%)	81	3	0	27
chr10	17168042	ywhaqa	CCCACCACATTCTTGTAGGCCAC	19/80 (23.8%)	81	3	0	18
chr19	12188165	ywhaqa	CCCACCACATTCTTGTAGGCCAC	19/80 (23.8%)	81	3	0	18
chr21	34112498	ywhaqa	CCAACCACGTTCTTGGAGGCCCA	19/80 (23.8%)	81	3	0	14

Supplementary Table S3. Information about all zebrafish samples examined in this study

Sample #	Sample ID	Gene targeted	Generation	Age	Fish per sample
1	F01p1_1	ldlra	F0	10 dpf	30
2	F01p1_2	ldlra	F0	10 dpf	30
3	F01p2_1	nbeal2	F0	10 dpf	25
4	F01p3_1	sh2b3	F0	10 dpf	30
5	F01p3_2	sh2b3	F0	10 dpf	30
6	F01p4_1	ywhaqa	F0	10 dpf	30
7	F01p4_2	ywhaqa	F0	10 dpf	1
8	F01s3_1	sh2b3	F0	adult	1
9	F01s3_2	sh2b3	F0	adult	1
10	F01s3_4	sh2b3	F0	adult	1
11	F01s3_5	sh2b3	F0	adult	1
12	F01s3_8	sh2b3	F0	adult	1
13	F01s3_9	sh2b3	F0	adult	1
14	F01s3_11	sh2b3	F0	adult	1
15	F01s3_12	sh2b3	F0	adult	1
16	F01s3_13	sh2b3	F0	adult	1
17	F01s3_15	sh2b3	F0	adult	1
18	F01s3_16	sh2b3	F0	adult	1
19	F01s4_1	ywhaqa	F0	adult	1
20	F01s4_2	ywhaqa	F0	adult	1
21	F01s4_3	ywhaqa	F0	adult	1
22	F01s4_4	ywhaqa	F0	adult	1
23	F01s4_5	ywhaqa	F0	adult	1
24	F01s4_6	ywhaqa	F0	adult	1
25	F01s4_7	ywhaqa	F0	adult	1
26	F01s4_8	ywhaqa	F0	adult	1
27	F01s4_9	ywhaqa	F0	adult	1
28	F01s4_10	ywhaqa	F0	adult	1
29	F01s4_11	ywhaqa	F0	adult	1
30	F01s4_12	ywhaqa	F0	adult	1
31	F01s4_14	ywhaqa	F0	adult	1
32	F01s4_15	ywhaqa	F0	adult	1
33	F01s4_16	ywhaqa	F0	adult	1
34	F03p1_1	ldlra	F0	5 dpf	30
35	F03p1_2	ldlra	F0	5 dpf	30
36	F03p1_3	ldlra	F0	5 dpf	30
37	F03p2_1	nbeal2	F0	5 dpf	30
38	F03p2_2	nbeal2	F0	5 dpf	30
39	F03p2_3	nbeal2	F0	5 dpf	30
40	F03p3_1	sh2b3	F0	5 dpf	30
41	F03p3_2	sh2b3	F0	5 dpf	30
42	F03p3_3	sh2b3	F0	5 dpf	30
43	F03p4_1	ywhaqa	F0	5 dpf	30
44	F03p4_2	ywhaqa	F0	5 dpf	30
45	F03p4_3	ywhaqa	F0	5 dpf	30
46	F12p3_1	sh2b3	F1	5 dpf	30
47	F12p3_2	sh2b3	F1	5 dpf	30
48	F12p3_3	sh2b3	F1	5 dpf	30
49	F12p3_4	sh2b3	F1	5 dpf	30

50	F12p3_5	sh2b3	F1	5 dpf	30
51	F12p3_6	sh2b3	F1	5 dpf	30
52	F12p3_7	sh2b3	F1	5 dpf	30
53	F12p4_1	ywhaqa	F1	5 dpf	30
54	F12p4_2	ywhaqa	F1	5 dpf	30
55	F12p4_3	ywhaqa	F1	5 dpf	30
56	F12p4_4	ywhaqa	F1	5 dpf	30
57	F12p4_5	ywhaqa	F1	5 dpf	30
58	F12p4_6	ywhaqa	F1	5 dpf	30
59	F12p4_7	ywhaqa	F1	5 dpf	30
60	F12p4_8	ywhaqa	F1	5 dpf	30
61	F12p4_9	ywhaqa	F1	5 dpf	30
62	F12s3_1	sh2b3	F1	juvenile	1
63	F12s3_2	sh2b3	F1	juvenile	1
64	F12s3_3	sh2b3	F1	juvenile	1
65	F12s3_4	sh2b3	F1	juvenile	1
66	F12s3_5	sh2b3	F1	juvenile	1
67	F12s3_6	sh2b3	F1	juvenile	1
68	F12s3_7	sh2b3	F1	juvenile	1
69	F12s3_8	sh2b3	F1	juvenile	1
70	F12s3_9	sh2b3	F1	juvenile	1
71	F12s3_10	sh2b3	F1	juvenile	1
72	F12s3_11	sh2b3	F1	juvenile	1
73	F12s3_12	sh2b3	F1	juvenile	1
74	F12s3_13	sh2b3	F1	juvenile	1
75	F12s3_14	sh2b3	F1	juvenile	1
76	F12s3_15	sh2b3	F1	juvenile	1
77	F12s3_16	sh2b3	F1	juvenile	1
78	F12s3_17	sh2b3	F1	juvenile	1
79	F12s3_18	sh2b3	F1	juvenile	1
80	F12s3_19	sh2b3	F1	juvenile	1
81	F12s3_20	sh2b3	F1	juvenile	1
82	F12s3_21	sh2b3	F1	juvenile	1
83	F12s3_22	sh2b3	F1	juvenile	1
84	F12s4_1	ywhaqa	F1	juvenile	1
85	F12s4_2	ywhaqa	F1	juvenile	1
86	F12s4_3	ywhaqa	F1	juvenile	1
87	F12s4_4	ywhaqa	F1	juvenile	1
88	F12s4_5	ywhaqa	F1	juvenile	1
89	F12s4_6	ywhaqa	F1	juvenile	1
90	F12s4_7	ywhaqa	F1	juvenile	1
91	F12s4_8	ywhaqa	F1	juvenile	1
92	F12s4_9	ywhaqa	F1	juvenile	1
93	F12s4_10	ywhaqa	F1	juvenile	1
94	F12s4_11	ywhaqa	F1	juvenile	1
95	F12s4_12	ywhaqa	F1	juvenile	1
96	F12s4_13	ywhaqa	F1	juvenile	1
97	F12s4_14	ywhaqa	F1	juvenile	1
98	F12s4_15	ywhaqa	F1	juvenile	1
99	F12s4_16	ywhaqa	F1	juvenile	1
100	F12s4_17	ywhaqa	F1	juvenile	1
101	F12s4_18	ywhaqa	F1	juvenile	1
102	F12s4_19	ywhaqa	F1	juvenile	1
103	F12s4_20	ywhaqa	F1	juvenile	1

104	F12s4_21	ywhaqa	F1	juvenile	1
105	F12s4_22	ywhaqa	F1	juvenile	1
106	F12s4_23	ywhaqa	F1	juvenile	1
107	F12s4_24	ywhaqa	F1	juvenile	1
108	F01wt_01	uninjected	F0	10 dpf	30
109	wtF0_1-5A	uninjected	F0	juvenile	5

Supplementary Table S4. Primer information for long-read amplicon re-sequencing of on- and off-target sites

ID	Forward primer	Reverse Primer	Forward primer	Reverse Primer	Expected size (bp)
ldlra on-target	GGACTGATCTGCCTCTGAATG	CACTTCCAGCTACCGTGTATG	chr3:19,302,116-19,302,136	chr3:19,308,406-19,308,426	6290
ldlra off-target 1	TGGAGGTCTGGTTGCTCTAA	GTGTATGGAGAGAGATGGGTTG	chr23:8,186,939-8,186,958	chr23:8,192,392-8,192,412	5453
ldlra off-target 2	ACGCACACATACCAGTCATC	CACACAGCTAACAACCCTACA	chr16:9,327,571-9,327,590	chr16:9,333,214-9,333,234	5664
ldlra off-target 3	CCAGAGTAATTGGCAGGTCTAC	GATCGAGCACGCTGTTCTTA	chr10:36,348,775-36,348,796	chr10:36,354,172-36,354,191	5417
ldlra off-target 4	TCTGCGTGTGAGAGATTGTATG	GCTACCCTTGGTGGTGTAAA	chr19:25,974,714-25,974,735	chr19:25,980,375-25,980,394	5661
ldlra off-target 5	AACAGACGAGCAGTTGGTATAG	CATAGTAGCCGAGAGGTGTTTC	chr24:7,923,642-7,923,663	chr24:7,926,264-7,926,285	2622
nbeal2 on-target	GAGATGGGTGAATGGAAGGATAA	TCTTGGATGACCTAAGGGTAGA	chr16:16,474,916-16,474,938	chr16:16,481,105-16,481,126	6211
nbeal2 off-target 1	CGATGGTAGGTGGGTTTCTATC	GGTGAATGGCGCAGTTTATTT	chr12:24,369,712-24,369,733	chr12:24,375,091-24,375,111	5400
nbeal2 off-target 2	CCTACTCTCTGCCCAAGATAAAG	CGGCTTCAACCACAACATTC	chr8:47,071,177-47,071,199	chr8:47,076,345-47,076,364	5188
nbeal2 off-target 3	TCAGCATAGCAGCAGCATAG	CTCGGTAGGTCAACACAAGTAG	chr14:16,381,452-16,381,471	chr14:16,386,500-16,386,521	5070
nbeal2 off-target 6	CCCGCTTGCCTCTTCATATT	CAGAGTGTAGGTTGGCCATTT	chr18:14,201,917-14,201,936	chr18:14,208,077-14,208,097	6181
nbeal2 off-target 7	CAGAGCCTGAGATGGAAGATATG	GATGTGTCGTACGGGTCAAA	chr10:1,888,407-1,888,429	chr10:1,893,485-1,893,504	5098
nbeal2 off-target 8	GCTACAAACTCTGTCTCCTCTTC	GCGAGTAATGGTTCCTGTAACT	chr3:35,682,090-35,682,112	chr3:35,687,058-35,687,079	4990
nbeal2 off-target 9	GTCACAAGCCCTCCACTAAA	GCTCCTCTCACTCAGGATTAAC	chr20:24,395,348-24,395,367	chr20:24,400,556-24,400,577	5230
nbeal2 off-target 10	GCAACTCTCGAGTCACTTTCT	AGCGGTTATGGTCGCATTAT	chr6:23,358,162-23,358,182	chr6:23,364,462-23,364,481	6320
nbeal2 off-target 11	GCCTGTGCTCATACGATTCT	CACTGTAAGCCTCTGACACTATT	chr14:11,137,744-11,137,763	chr14:11,143,443-11,143,465	5722
nbeal2 off-target 12	GCGACCCAGAAGTCTCTTTAC	CCTTCGCTCTCTCTTTCATCTC	chr18:30,357,214-30,357,234	chr18:30,362,099-30,362,120	4907
sh2b3 on-target	GAGATCCGAACTGCTGGATTTA	GTTTGGCTTGCTGAGCTTATG	chr5:9,621,342-9,621,363	chr5:9,626,092-9,626,112	4771
sh2b3 off-target 1	CTCTTGCTATTTCGGGCTAGTG	GTGTCGTGATGTCTCTGTCTTG	chr15:31,594,333-31,594,354	chr15:31,601,209-31,601,230	6898
sh2b3 off-target 2	GCAGAACCTCGTGATGTCTATC	CGCTGGTCCACTTCTCAATTA	chr10:20,536,920-20,536,941	chr10:20,543,191-20,543,211	6292

ywhaqa on-target	CTCTTTCCGCTCCCTATGAATG	GTGATGAGGTGCCAAGCTATAA	chr20:29,564,081-29,564,102	chr20:29,570,097-29,570,118	6038
ywhaqa off-target 1	TTCACGCTACCTCTGTTCTTG	CGTGCAGCCCTATAGTTCTAAT	chr5:3,540,188-3,540,208	chr5:3,547,891-3,547,912	7703
ywhaqa off-target 2	CATCGGTGTGCTAAACTGAAATG	GTCTCTCTGCGAACAAGGTAAA	chr17:32,501,047-32,501,069	chr17:32,506,771-32,506,792	5746
ywhaqa off-target 3	GAAGTGGTACTGCCCTGAATAC	TGCGTCGACACAACCTAATC	chr6:52,212,611-52,212,632	chr6:52,219,045-52,219,064	6454
ywhaqa off-target 4	CGACACTCAGGTAAGAGAACAC	AGGACCAGAAGATGAAGGTAGA	chr16:24,681,274-24,681,295	chr16:24,685,847-24,685,867	4594
ywhaqa off-target 5	GACCTCATCATTGGCCCTAAA	GCAGAAGCACTGCAACATAAA	chr10:17,165,822-17,165,842	chr10:17,170,415-17,170,435	4614
ywhaqa off-target 6	TTACTCGTCCCAACTGCTATTT	GGTCTGGGATACGGTTTGTT	chr19:12,184,966-12,184,987	chr19:12,190,849-12,190,868	5903
ywhaqa off-target 7	GGCCAAAGATGGAGGATATGAG	GCGTAGAGCGCGATAGTTAAT	chr21:34,109,119-34,109,140	chr21:34,115,564-34,115,584	6466

Supplementary Table S5. Distinct CRISPR-Cas9 alleles detected at *sh2b3* on-target, both in F1 individuals and F1 larvae pools, obtained from *sh2b3* founder pair #1.

Allele	Variant	Del size (bp)	Ins size (bp)
1	INSERTION	0	8
2	DELINS	3	6
3	DELETION	5	0
4	DELINS	6	5
5	DELETION	9	0

Supplementary Table S6. Distinct CRISPR-Cas9 alleles detected at *sh2b3* on-target, both in F1 individuals and F1 larvae pools, obtained from *sh2b3* founder pair #2.

Allele	Variant	Del size (bp)	Ins size (bp)
1	DELETION	6	0
2	DELETION	11	0
3	DELINS	1	5
4	DELINS	15	6

Supplementary Table S7. Distinct CRISPR-Cas9 alleles detected at *sh2b3* on-target, both in F1 individuals and F1 larvae pools, obtained from *sh2b3* founder pair #3.

Allele	Variant	Del size (bp)	Ins size (bp)
1	DELETION	6	0
2	DELETION	12	0
3	DELETION	10	0
4	DELINS	2	9
5	DELETION	9	0

Supplementary Table S8. Distinct CRISPR-Cas9 alleles detected at *ywhaqa* on-target, both in F1 individuals and F1 larvae pools, obtained from *ywhaqa* founder pair #1.

Allele	Variant	Del size (bp)	Ins size (bp)
1	INSERTION	0	2
2	DELETION	21	0
3	DELETION	2	0
4	DELETION	6	0

Supplementary Table S9. Distinct CRISPR-Cas9 alleles detected at *ywhaqa* on-target, both in F1 individuals and F1 larvae pools, obtained from *ywhaqa* founder pair #2.

Allele	Variant	Del size (bp)	Ins size (bp)
1	INSERTION	0	4
2	DELETION	3	0
3	DELINS	1	292
4	DELETION	6	0
5	DELETION	13	0
6	DELINS	381	22

Supplementary Table S10. Distinct CRISPR-Cas9 alleles detected at *ywhaqa* on-target, in F1 individuals and F1 larvae pools, obtained from *ywhaqa* founder pair #4.

Allele*	Variant	Del size (bp)	Ins size (bp)
1	DEL	6	0
2	DELINS	4	6

*Only one F1 larvae pool was available for this founding pair

Supplementary Table S11. List of off-target events and large SVs in the F1 individuals, which are validated in F1 zebrafish larvae pools obtained from the same mating pair.

Site	Sample	Variant	Freq	Mating pair	Matched pools	Validated in IGV	Validated in pool
sh2b3 on-target	F12s3_7	1053 bp del	0.9728261	pair 3	F12p3_3, F12p3_4, F12p3_6	YES	YES
sh2b3 off-target 1	F12s3_1	8 bp del	0.7214854	pair 2	F12p3_7	YES	YES
sh2b3 off-target 1	F12s3_4	8 bp del	0.7152445	pair 2	F12p3_7	YES	YES
sh2b3 off-target 1	F12s3_5	8 bp del	0.7163842	pair 2	F12p3_7	YES	YES
sh2b3 off-target 1	F12s3_7	5 bp ins	0.5784615	pair 3	F12p3_3, F12p3_4, F12p3_6	YES	YES
sh2b3 off-target 1	F12s3_9	8 bp del	0.7235974	pair 2	F12p3_7	YES	YES
sh2b3 off-target 1	F12s3_10	8 bp del	0.7313567	pair 2	F12p3_7	YES	YES
ywhaqa on-target	F12s4_20	292 bp ins	0.8678611	pair 2	F12p4_4, F12p4_5, F12p4_6	YES	YES
ywhaqa on-target	F12s4_22	292 bp ins	0.9976162	pair 2	F12p4_4, F12p4_5, F12p4_6	YES	YES
ywhaqa on-target	F12s4_23	292 bp ins	0.9773414	pair 2	F12p4_4, F12p4_5, F12p4_6	YES	YES
ywhaqa off-target 2	F12s4_9	2 bp del	0.2772384	pair 4	F12p4_9	YES	YES
ywhaqa off-target 2	F12s4_17	3 bp del	0.8082902	pair 2	F12p4_4, F12p4_5, F12p4_6	YES	YES
ywhaqa off-target 2	F12s4_20	3 bp del	0.6814159	pair 2	F12p4_4, F12p4_5, F12p4_6	YES	YES
ywhaqa off-target 2	F12s4_21	3 bp del	0.8082139	pair 2	F12p4_4, F12p4_5, F12p4_6	YES	YES
ywhaqa off-target 2	F12s4_23	3 bp del	0.8852459	pair 2	F12p4_4, F12p4_5, F12p4_6	YES	YES
ywhaqa off-target 2	F12s4_24	3 bp del	0.4630463	pair 2	F12p4_4, F12p4_5, F12p4_6	YES	YES

Supplementary Table S12. List of on-target editing events in F1 individuals that are seemingly homozygous and where one single allele is reported in >98% of the sequencing reads.

Site	Sample name	Mating pair	Variant	Del size	Ins size	Validated in IGV
sh2b3 on-target	F12s3_18	pair 1	DELINS	6	5	YES
ywhaqa on-target	F12s4_8	pair 4	DELINS	4	6	YES
ywhaqa on-target	F12s4_9	pair 4	DELINS	4	6	YES
ywhaqa on-target	F12s4_12	pair 4	DELINS	4	6	YES
ywhaqa on-target	F12s4_16	pair 1	DELETION	6	0	YES
ywhaqa on-target	F12s4_17	pair 2	DELETION	3	0	YES
ywhaqa on-target	F12s4_18	pair 2	DELETION	6	0	YES

Supplementary Table S13. Structural variants detected by Sniffles in nanopore WGS data from *ywhaqa* F1 individual #5. Only SVs having a breakpoint in a 5kb window from the ywhaqa on-target site are listed in this table.

chr	pos	SV type	insertion sequence
20	29564831	41bp deletion	
20	29564866	66bp insertion	ATATATATATATATATATATATATATATATATATATATATATATATATATATATATATATATATAT
20	29567303	48bp deletion	

Supplementary Table S14. Structural variants detected by Sniffles in nanopore WGS data from *ywhaqa* F1 individual #17. Only SVs having a breakpoint in a 5kb window from the ywhaqa on-target site are listed in this table.

chr	pos	SV type	insertion sequence
20	29563718	559bp insertion	CAGGGCCGGAGTGGGACTCTTTTTCAGCCCTGGAGTTTCAGAAGCCTCAGACCGGCCC ACTCAGTTCACCACTGACTATATTAAAATAAGGTTATTTCCAATTCAGTTTCTAATTACACT ATCACGTCTTTTGAAAGCAACACTTTTAGAACTTCAAATGTTCAACAACCCATACAGTATT ATGTAACAATAAAAATGAAAAAAAAAAATTGTTACTCAGACAAGGACGAACCCAGGTTGG CCACGTCATAATCTAACGTACTAACCACTGTACCACAACAGCTGTAAGTATAATGGTGACT TATCTAATTATATCATCATTATCCTGCATCCTGCTCACGAGGCTACGAGAAAATATATAAAA GAGCAGAGCTGCGGTAAAATAATGAATAAGAAGGCTGTGGTCAAGTAAATAATTAAATT ATTTTTAAAAAGTGTGATGACTGCTGAAACCGATTCTGGAACTAGGGACACCGGCCCTC GCTGACAAAAAACGGACCGGCCCACCGGGAAATGCTCCCGGTCCTTCCGATTAGCCAA TCCGGGCCTGACCATGAA
20	29564831	41bp deletion	
20	29564866	65bp insertion	ATATATATATATATATATATATATATATATATATATATATATATATATATATATATATATATATAT
20	29567303	48bp deletion	
