# Supplementary material for: CRISPR-Cas9 induces large structural variants at on-target and off-target sites in vivo that segregate across generations
Source: Nat Commun. 2022 Feb 2;13:627. doi: 10.1038/s41467-022-28244-5 (PMC8810904; doi:10.1038/s41467-022-28244-5)
Supplement: Supplementary file 2 — Reporting Summary [file 41467_2022_28244_MOESM2_ESM.pdf]

nature portfolio

Corresponding author(s): Adam Ameur

Last updated by author(s): December 22, 2021

Reporting Summary

Nature Portfolio wishes to improve the reproducibility of the work that we publish. This form provides structure for consistency and transparency in reporting. For further information on Nature Portfolio policies, see our Editorial Policies and the Editorial Policy Checklist.

Statistics

For all statistical analyses, confirm that the following items are present in the figure legend, table legend, main text, or Methods section.

n/a	Confirmed	
☒	☐	The exact sample size (*n*) for each experimental group/condition, given as a discrete number and unit of measurement
☒	☐	A statement on whether measurements were taken from distinct samples or whether the same sample was measured repeatedly
☒	☐	The statistical test(s) used AND whether they are one- or two-sided<br>*Only common tests should be described solely by name; describe more complex techniques in the Methods section.*
☒	☐	A description of all covariates tested
☒	☐	A description of any assumptions or corrections, such as tests of normality and adjustment for multiple comparisons
☒	☐	A full description of the statistical parameters including central tendency (e.g. means) or other basic estimates (e.g. regression coefficient) AND variation (e.g. standard deviation) or associated estimates of uncertainty (e.g. confidence intervals)
☒	☐	For null hypothesis testing, the test statistic (e.g. *F*, *t*, *r*) with confidence intervals, effect sizes, degrees of freedom and *P* value noted<br>*Give P values as exact values whenever suitable.*
☒	☐	For Bayesian analysis, information on the choice of priors and Markov chain Monte Carlo settings
☒	☐	For hierarchical and complex designs, identification of the appropriate level for tests and full reporting of outcomes
☒	☐	Estimates of effect sizes (e.g. Cohen's *d*, Pearson's *r*), indicating how they were calculated

Our web collection on statistics for biologists contains articles on many of the points above.

Software and code

Policy information about availability of computer code

Data collection	SMRTLink v10.1, Guppy v4.0 & v5.0
Data analysis	Minimap2 v2.18, Insider v1.8 (https://github.com/UppsalaGenomeCenter/InSiDeR), EMBOSS-Needle v6.6.0, SIQ v1.0 (https://github.com/RobinVanSchendel/SIQ), NGMLR v0.2.7, Sniffles v1.0.12, bamCoverage v3.3.2, deepTools v3.3.2

For manuscripts utilizing custom algorithms or software that are central to the research but not yet described in published literature, software must be made available to editors and reviewers. We strongly encourage code deposition in a community repository (e.g. GitHub). See the Nature Portfolio guidelines for submitting code & software for further information.

Data

Policy information about availability of data

All manuscripts must include a data availability statement. This statement should provide the following information, where applicable:
- Accession codes, unique identifiers, or web links for publicly available datasets
- A description of any restrictions on data availability
- For clinical datasets or third party data, please ensure that the statement adheres to our policy

The sequence data that support the findings of this study have been deposited in the NCBI Sequence Read Archive (SRA) with the accession code PRJNA772901 (https://www.ncbi.nlm.nih.gov/Traces/study/?acc=PRJNA772901). Source data are provided with this paper. The GRCz11 reference genome used in this study is available in the file “danRer11.fa.gz” from https://hgdownload.soe.ucsc.edu/goldenPath/danRer11/bigZips/.

Field-specific reporting

Please select the one below that is the best fit for your research. If you are not sure, read the appropriate sections before making your selection.

☒ Life sciences ☐ Behavioural & social sciences ☐ Ecological, evolutionary & environmental sciences

For a reference copy of the document with all sections, see nature.com/documents/nr-reporting-summary-flat.pdf

Life sciences study design

All studies must disclose on these points even when the disclosure is negative.

Sample size	No sample size calculation was performed. DNA from over 1100 zebrafish was analyzed in this study, from larvae pools and from individual zebrafish. With this sample size, it is possible to identify commonly occurring CRISPR-Cas9 induced events, but also rare events affecting a low percentage of individuals (10% or less).
Data exclusions	Samples that failed during the library preparation, or failed to produce sequencing coverage of the targeted sites, were excluded from the data set
Replication	Three replicate CRISPR-Cas9 genome editing and crossing experiments were performed consecutively, using fertilized eggs from different parents of the same zebrafish line (ABs). All three replicate genome editing experiments were successful. Several samples were analyzed at each developmental stage.
Randomization	Zebrafish in-crossing was performed by random selection of mating pairs. The allocation of zebrafish into control or editing groups was done at random, as well as the collection of zebrafish larvae into larger pools
Blinding	Zebrafish husbandry was performed under identical conditions, blinding was not used

Reporting for specific materials, systems and methods

We require information from authors about some types of materials, experimental systems and methods used in many studies. Here, indicate whether each material, system or method listed is relevant to your study. If you are not sure if a list item applies to your research, read the appropriate section before selecting a response.

Materials & experimental systems

n/a	Involved in the study
☒	☐ Antibodies
☒	☐ Eukaryotic cell lines
☒	☐ Palaeontology and archaeology
☐	☒ Animals and other organisms
☒	☐ Human research participants
☒	☐ Clinical data
☒	☐ Dual use research of concern

Methods

n/a	Involved in the study
☒	☐ ChIP-seq
☒	☐ Flow cytometry
☒	☐ MRI-based neuroimaging

Animals and other organisms

Policy information about studies involving animals; ARRIVE guidelines recommended for reporting animal research

Laboratory animals	Zebrafish (Danio rerio) used in this study originate from the AB strain. Samples were collected at the larval stage (5 or 10 days post-fertilization) as well as when founder fish reached adulthood (3 months). In the F1 generation, samples were collected at the larval stage as well as from juvenile fish (2 months). Zebrafish do not have sex chromosomes and sex differentiation takes place between day 20 and 25 post-fertilization. This implies that the larvae, by definition, are neither male nor female. No sex information was collected for the juvenile or adult zebrafish.
Wild animals	This study does not involve wild animals
Field-collected samples	This study does not involve samples collected in the field
Ethics oversight	The zebrafish experiments have been approved by the Uppsala University Ethical Committee for Animal Research (Dnr 5.8.18-13680/2020).

Note that full information on the approval of the study protocol must also be provided in the manuscript.
